# Editorial: Targeting α-Synuclein in Parkinson's Disease and Multiple System Atrophy

**DOI:** 10.3389/fneur.2022.942313

**Published:** 2022-06-10

**Authors:** Lisa Fellner, Franziska Richter, Patrik Brundin, Johannes Haybaeck

**Affiliations:** ^1^Department of Genomics, Stem Cell Biology and Regenerative Medicine, Institute of Molecular Biology and Center for Molecular Biosciences Innsbruck, Leopold-Franzens-University Innsbruck, Innsbruck, Austria; ^2^Department of Pharmacology, Toxicology and Pharmacy, University of Veterinary Medicine Hannover, Hanover, Germany; ^3^Center for Systems Neuroscience, Hanover, Germany; ^4^Center for Neurodegenerative Science, Van Andel Institute, Grand Rapids, MI, United States; ^5^Pharma Research and Early Development (pRED), F Hoffman-La Roche, Little Falls, NJ, United States; ^6^Institute of Pathology, Neuropathology and Molecular Pathology, Medical University of Innsbruck, Innsbruck, Austria; ^7^Diagnostic and Research Center for Molecular Biomedicine, Institute of Pathology, Medical University of Graz, Graz, Austria

**Keywords:** α-synuclein, multiple system atrophy (MSA), Parkinson's disease (PD), Lewy bodies (LBs), glial cytoplasmic inclusions (GCIs)

Parkinson's disease (PD) and multiple system atrophy (MSA) are progressive diseases that clinically manifest with motor (e.g., parkinsonism) and non-motor (e.g., autonomic failure) symptoms. PD and MSA belong to the devastating neurodegenerative disorders known as α-synucleinopathies, as both exhibit abnormal deposits of α-synuclein (α-syn) in the cytoplasm of cells. PD exhibits the inclusions primarily in neurons (Lewy bodies), whereas glial cytoplasmic inclusions are most common in MSA. It is not clear why α-syn handling is dysregulated and constitutes the main component of these pathological aggregates. Another unresolved issue is why α-syn inclusions predominantly occur in neurons in PD and in oligodendroglial cells in MSA. It is believed that the aberrant accumulation of α-syn leads to disruption of neuronal homeostasis, gliosis and neuronal cell death. Furthermore, it has been proposed that α-syn has properties of a prion-like protein, explaining why α-syn aggregates can propagate from one neuron to another and seed aggregation of α-syn in the cells that they enter. Currently, no effective treatment to stop the progression of these diseases is available. Different therapeutic approaches targeting α-syn are emerging, including e.g., immunotherapy, approaches to reduce α-syn expression and inhibitors of α-syn aggregation.

In the current Research Topic, Menon et al. present an overview on different treatment options to reduce or silence the expression of α-syn as well as to diminish the levels of substrate. This group describes the various approaches to stopping the spread of α-syn pathology, including active and passive immunization. Furthermore, the authors discuss the possibility of transiently enhancing the permeability of the blood-brain-barrier for increased brain delivery of anti-α-syn agents. An original article by Pagano et al. describes the safety and efficacy of prasinezumab, which is a humanized monoclonal antibody that binds aggregated α-syn and has been tested in patients with early PD (PASADENA, phase II). The authors summarize the rationale, design of this immunotherapy, and the baseline data of the PASADENA cohort.

To be able to effectively develop new therapeutic approaches to reduce the accumulation of misfolded α-syn, it is important to have experimental *in vitro* and *in vivo* models that faithfully mimic the pathomechanisms of α-synucleinopathies. In an original publication, Moudio et al. present an organotypic slice culture model to monitor α-syn aggregation. The authors could show that they can mimic several features of α-synucleinopathies, including cellular toxicity, mitochondrial dysfunction, autophagy activation and cell death. Thus, their model can serve as a platform for screening and testing disease-modifying therapeutic targets. In another original study, Höllerhage et al. explore LUHMES cells (a human midbrain dopamine neuron cell line) that overexpress α-syn as a PD model paradigm. The authors conduct transcriptome and proteome expression analysis and describe differential expression of 21 genes and corresponding proteins. Especially, genes involved in cell death and apoptotic signaling pathway were up-regulated in cells overexpressing α-syn, and most differentially regulated proteins are associated to the lysosome. The overlap with pathomechanisms (e.g., lysosomal dysfunction) is believed to be central to the pathogenesis of PD, further validates LUHMES cells as versatile cellular PD model.

Patient-derived inducible pluripotent stem cells (iPSC) self-renew indefinitely and can be differentiated into tissues belonging to all three germ layers. By carrying the genetic background of the patient, iPSC can potentially be valuable tools to study pathogenetic mechanisms. In a review in the current Research Topic, Spathopoulou et al. give an overview on PD models that are based on iPSC. They summarize studies using iPSC-derived 2D neuronal and 3D brain models carrying genetic PD-variants of α-syn. They conclude that patient-derived iPSC can be valuable tools to study α-syn-dependent disruptions of neuronal integrity.

Several studies have described that α-syn conformers can behave as distinct strains which impose their own specific fibrillar structures on α-syn monomers, inducing them to also assemble into similar fibrils. The mini review by Malfertheiner et al. discusses that the distinct α-syn strains might lead to different disease phenotypes, and that the existence of different strains enables the development of diagnostic biomarkers.

The α-syn aggregates seen in PD and MSA also accumulate other cellular components including proteins (e.g., ubiquitin) and lipids. The role of lipid dyshomeostasis in α-synucleinopathies is discussed by Bell and Vendruscolo in the current Research Topic. They describe that lipid membranes can promote the aggregation of α-syn. Furthermore, the authors highlight how post-translational modifications of α-syn can modulate the binding of α-syn to lipid membranes, further affecting the aggregation process.

Several studies have suggested that cytosolic Ca^2+^ is involved in the pathogenesis of α-synucleinopathies. This was part of the scientific premise for the use of isradipine, a CaV1.3 channel antagonist, in clinical trials aimed at slowing the progression of PD. Unfortunately, isradipine failed to slow disease progression in early-stage PD patients. The role of Ca^2+^ in the pathogenesis of PD and other synucleinopathies is discussed in an extensive review by Kovacs et al. The authors explain different ways, e.g., Ca^2+^ poor buffering capacity of dopaminergic neurons, in which elevated cytosolic Ca^2+^ might be involved in the progression of the disease. They also discuss a new Ca^2+^ hypothesis that is based on the interaction between Ca^2+^ and progressive α-syn aggregate toxicity in neurons.

Overall, the current Research Topic reminds us that the role of α-syn in neurodegenerative diseases is still not well-understood. A multitude of factors appear to interact and play a role in the initiation and progression of α-synucleinopathies. It is extremely important that future research identifies the key pathways and put them into disease context, for example explaining when during the progressive neurodegenerative process, they are prominent. We thank all the authors for their outstanding contributions to shed light on the complexity of α-synucleinopathies. We hope that they will help to garner significant progress in the field.

## Author Contributions

LF drafted the manuscript. FR, PB, and JH revised the manuscript. All authors read and approved the final manuscript.

## Conflict of Interest

FR is holding research contracts with Idorsia and Roche. PB has consulted for Axial Therapeutics, Calico, CuraSen, Enterin Inc, Fujifilm-Cellular Dynamics Inc, Idorsia, and Lundbeck A/S, has received commercial support for research from Lundbeck A/S and Roche, and has ownership interests in Acousort AB, Axial Therapeutics, Enterin Inc, and RYNE Biotechnology Inc. During the compilation of this special issue PB became an employee of F Hoffman-La Roche. The remaining authors declare that the research was conducted in the absence of any commercial or financial relationships that could be construed as a potential conflict of interest.

## Publisher's Note

All claims expressed in this article are solely those of the authors and do not necessarily represent those of their affiliated organizations, or those of the publisher, the editors and the reviewers. Any product that may be evaluated in this article, or claim that may be made by its manufacturer, is not guaranteed or endorsed by the publisher.

